# Transmission dynamics of seasonal influenza in a remote island population

**DOI:** 10.1038/s41598-023-32537-0

**Published:** 2023-04-03

**Authors:** Su Myat Han, Alexis Robert, Shingo Masuda, Takahiro Yasaka, Satoshi Kanda, Kazuhiri Komori, Nobuo Saito, Motoi Suzuki, Akira Endo, Marc Baguelin, Koya Ariyoshi

**Affiliations:** 1 School of Tropical Medicine and Global Health, Nagasaki University, Nagasaki, Japan; 2 Department of Infectious Disease Epidemiology, Faculty of Epidemiology and Population Health, London School of Hygiene and Tropical Medicine, London, UK; 3 Centre for the Mathematical Modelling of Infectious Diseases, London School of Hygiene & Tropical Medicine, Keppel Street, London, UK; 4 Department of Internal Medicine, Kamigoto Hospital, Kamigoto, Japan; 5 Department of Microbiology, Faculty of Medicine, Oita University, Yufu, Japan; 6 Department of Clinical Medicine, Institute of Tropical Medicine, Nagasaki University, Nagasaki, Japan; 7 Infectious Disease Surveillance Center, National Institute of Infectious Diseases, Tokyo, Japan; 8 MRC Centre for Global Infectious Disease Analysis and the Abdul Latif Jameel Institute for Disease, London, UK

**Keywords:** Ecological epidemiology, Risk factors, Population dynamics, Disease prevention, Health policy, Public health

## Abstract

Seasonal influenza outbreaks remain an important public health concern, causing large numbers of hospitalizations and deaths among high-risk groups. Understanding the dynamics of individual transmission is crucial to design effective control measures and ultimately reduce the burden caused by influenza outbreaks. In this study, we analyzed surveillance data from Kamigoto Island, Japan, a semi-isolated island population, to identify the drivers of influenza transmission during outbreaks. We used rapid influenza diagnostic test (RDT)-confirmed surveillance data from Kamigoto island, Japan and estimated age-specific influenza relative illness ratios (RIRs) over eight epidemic seasons (2010/11 to 2017/18). We reconstructed the probabilistic transmission trees (i.e., a network of who-infected-whom) using Bayesian inference with Markov-chain Monte Carlo method and then performed a negative binomial regression on the inferred transmission trees to identify the factors associated with onwards transmission risk. Pre-school and school-aged children were most at risk of getting infected with influenza, with RIRs values consistently above one. The maximal RIR values were 5.99 (95% CI 5.23, 6.78) in the 7–12 aged-group and 5.68 (95%CI 4.59, 6.99) in the 4–6 aged-group in 2011/12. The transmission tree reconstruction suggested that the number of imported cases were consistently higher in the most populated and busy districts (Tainoura-go and Arikawa-go) ranged from 10–20 to 30–36 imported cases per season. The number of secondary cases generated by each case were also higher in these districts, which had the highest individual reproduction number (R_eff_: 1.2–1.7) across the seasons. Across all inferred transmission trees, the regression analysis showed that cases reported in districts with lower local vaccination coverage (incidence rate ratio IRR = 1.45 (95% CI 1.02, 2.05)) or higher number of inhabitants (IRR = 2.00 (95% CI 1.89, 2.12)) caused more secondary transmissions. Being younger than 18 years old (IRR = 1.38 (95%CI 1.21, 1.57) among 4–6 years old and 1.45 (95% CI 1.33, 1.59) 7–12 years old) and infection with influenza type A (type B IRR = 0.83 (95% CI 0.77, 0.90)) were also associated with higher numbers of onwards transmissions. However, conditional on being infected, we did not find any association between individual vaccination status and onwards transmissibility. Our study showed the importance of focusing public health efforts on achieving high vaccine coverage throughout the island, especially in more populated districts. The strong association between local vaccine coverage (including neighboring regions), and the risk of transmission indicate the importance of achieving homogeneously high vaccine coverage. The individual vaccine status may not prevent onwards transmission, though it may reduce the severity of infection.

## Introduction

Seasonal influenza remains a major public health threat globally with a substantial economic and health care burden each year^1^. It is estimated that about 3–5 million people suffer severe illness due to seasonal influenza, and influenza-associated infections are estimated to cause between 290,000 and 650,000 deaths every year (WHO)^2^. It is therefore a key to understand the drivers of influenza transmission and identify the interventions able to reduce the burden of influenza.

Empirical and modeling studies have revealed a number of key characteristics of the spatial transmission of seasonal influenza over multiple seasons^3–13^. The dynamics of human mobility patterns played a key role resulting in hierarchical transmission of influenza with short commuting responsible for wave-like transmission on a small geographical scale, while air travels was responsible for long-range seedings behaviors^5,8,12^. Seasonality, dominating influenza strain in each epidemic, humidity and temperature are also reported to affect the spatial dynamics of influenza^10,14–16^. These studies were conducted at a global, regional, or country level, with very limited representation to the transmission dynamics at smaller geographical levels, such as islands or cities. Analysis conducted at a smaller geographical granularity in areas with limited incoming transportation links may capture unique patterns of disease transmission that would not be visible in aggregated data at a larger scale.

We studied patterns of influenza transmission using the unique fine scale linelist dataset collected in Kamigoto island, Japan. Kamigoto island is located on the western coast of Nagasaki prefecture in Japan, semi-isolated from mainland Japan due to limited transportation links. The setting is therefore ideal for understanding the driving force behind local transmission without being severely affected by interferences from other regions or long-distance mobility. We first calculated the relative illness ratios and attack rates between age groups to identify groups at greatest risk of infection. Secondly, we explored what factors were associated with the risk of onwards transmission. To do so, we inferred the network of who-infected-whom (i.e., probabilistic transmission trees) using a Bayesian approach, which resulted in a set of probabilistic transmission trees that we used to estimate the likely number of secondary cases caused by each case in our dataset. We then performed a negative binomial regression analysis on each inferred transmission network to identify factors associated with increased risk of onwards transmission.

## Methods

### Study setting

This study is a retrospective observational study over eight influenza seasons (2010/11 to 2017/18 seasons) in Kamigoto island. Kamigoto island is located on the western coast of Nagasaki prefecture, Japan. Kamigoto island has 39 districts, with approximately 22,000 residents in total. Population sizes of the districts range from 40 to over 3000 inhabitants. The island is semi-isolated due to its limited transportation links to mainland Japan. There is only one hospital within the island, which most of influenza-like illness (ILI) cases attended for the diagnosis of influenza during the study period. Aside from the hospital, there are six clinics on the island. Influenza-like cases and rapid diagnostic test (RDT)-confirmed influenza cases within the island were monitored by the ILI registry system as influenza surveillance. In Japan, influenza vaccination is recommended annually between late October to December. Routine vaccination is recommended to people aged 65 and above, and for high-risk population aged 60 and above. For children, influenza vaccination is not under compulsory vaccination according to the national immunization schedule of Japan.

### Data source

The study dataset is based on surveillance data and contains information on all patients who visited Kamigoto Hospital with RDT-confirmed influenza between October 2010 and April 2018 (8497 patients in total). In Japan, ILI and influenza had been under systematic surveillance following the new infectious disease control law effective since April 1999^17^. An ILI case is defined as the sudden onset of fever over 38 °C, respiratory symptoms, and other systemic symptoms (fatigue, headache, or myalgia)^18^. All individuals with ILI symptoms visiting the hospital during influenza season were tested with a commercial RDT to diagnose influenza A- and B-positive cases as a routine practice. RDTs are widely used in Japan and can serve as a proxy of the total symptomatic influenza case in the population^18^. Sensitivity of RDTs in Japan is estimated to be relatively high: 72.9–96.4% across all the studies conducted in Japan^19,20^. Previous studies suggested that the reporting ratio of ILI and influenza cases were higher in Japan compared to other countries due to the wide use of RDTs^21^. In our dataset, 107 cases were not RDT positive but clinically diagnosed influenza, and only one case was coinfected (A + B). Cases with the history of travelling into/outside the island within 7 days prior to the onset were identified as import cases in the surveillance system. A total of 959 cases (11.2% of all the RDT-confirmed influenza cases) were recorded as epidemiologically identified imported cases.

**Figure 1:**
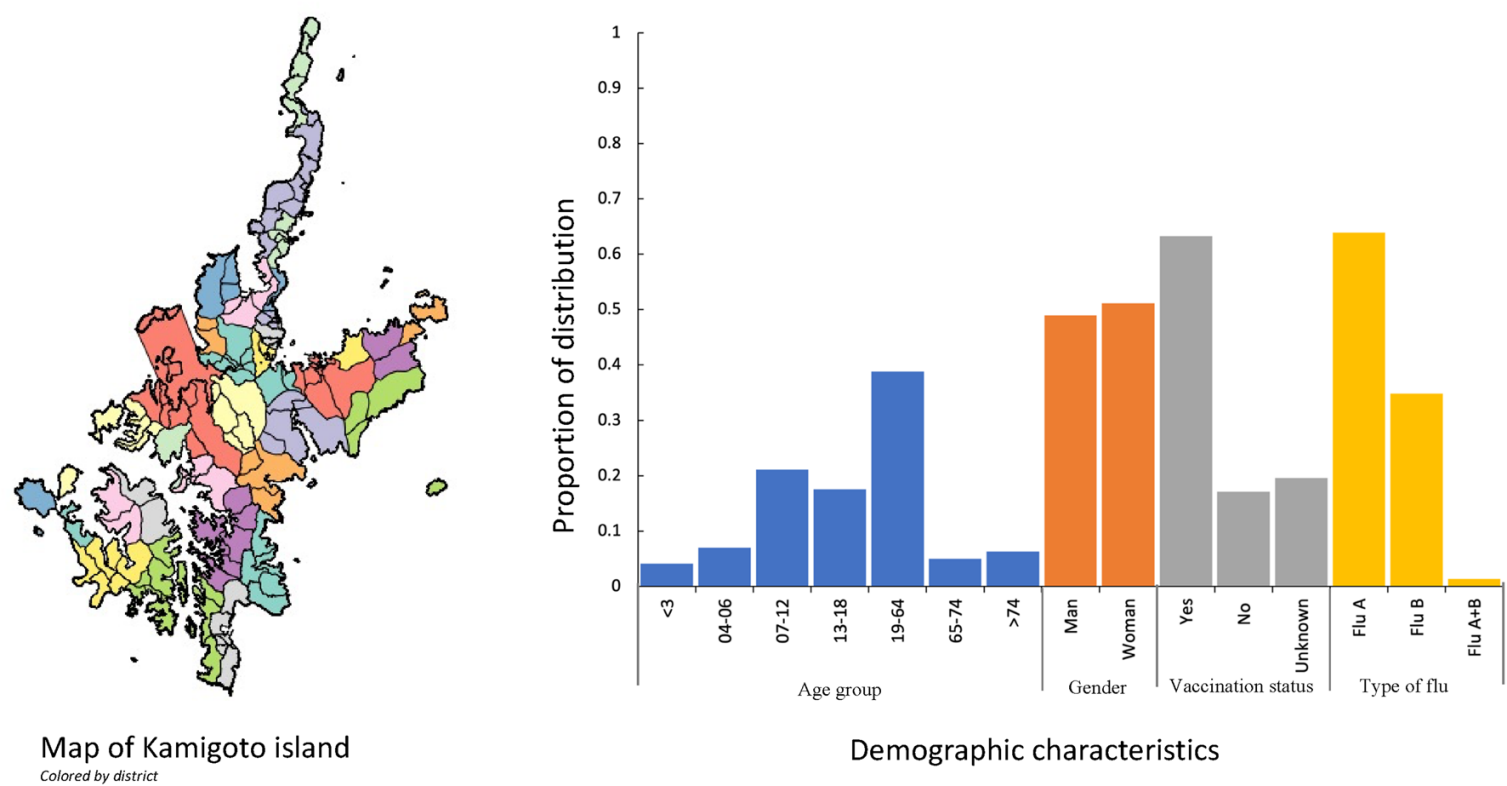
Map of Kamigoto island (each color represents a district), and demographic characteristics of the surveillance dataset of influenza, Kamigoto Island, Japan 2010/11–2017/18 influenza season.

The surveillance dataset contains the age, sex, date of onset, date and result of RDT test of each case. In addition, the local health authorities and government collected data on influenza vaccination statuses, distribution of the age group of residents, influenza vaccination coverage by district and by age group in each season via the vaccination record system for the entire population on the island. Individuals were classified as ‘vaccinated’ if they were 14 days past vaccine administration. Postcode of residence was collected for influenza positive cases^22^. In the dataset, 134 cases (1.6% of total cases) had no post-code information recorded and thus were excluded in the reconstruction of transmission analysis. Demographic characteristics of the cases are presented in Fig. 1.

### Relative illness ratio (RIR)

To identify the burden of age groups most infected by influenza, first we calculated the relative illness ratio (RIR). RIR is defined as the ratio of the percentage of influenza cases in age group *i* (seven age groups in this study: below 3 years old; 4–6; 7–12; 13–18; 19–64; 65–74; above 75 years old), to the percentage of the total population belonging to that *i*th age-group. The formula as follows.

$$RIR_{i} = \frac{{{{C_{i} } \mathord{\left/ {\vphantom {{C_{i} } {\sum\nolimits_{j = 1}^{n} {C_{j} } }}} \right. \kern-0pt} {\sum\nolimits_{j = 1}^{n} {C_{j} } }}}}{{{{N_{i} } \mathord{\left/ {\vphantom {{N_{i} } {\sum\nolimits_{j = 1}^{n} {N_{j} } }}} \right. \kern-0pt} {\sum\nolimits_{j = 1}^{n} {N_{j} } }}}}$$

${C}_{i}$; number of influenza cases in age group *i* (n age groups), ${N}_{i}$; population size of age group *i*, *C*_*j*_; total number of influenza cases, *N*_*j*_; total population size.

RIR values above 1 indicate excess risk of infection. Confidence interval (CIs) were calculated with the Poisson exact method^23–25^. More details about age-standardized relative illness ratio (RIR) were described in previous studies^23–25^.

### Probabilistic reconstruction of transmission chains

In order to highlight determinants frequently associated with onward transmission, we had to reconstruct who-infected-whom during influenza outbreaks reported on Kamigoto Island. Transmission history can be reconstructed through contact-tracing investigations, where infected cases are asked who they had been in contact with over their infectious period. However, such contact-tracing investigations were not carried out as part of flu surveillance on Kamigoto island. Therefore, we needed an inference framework to estimate who-infected-whom using features available in the case data.

Transmission tree reconstruction methods often require genetic sequences, and group cases according to how closely they are genetically related. However, we did not have access to genetic sequences in this analysis, only to epidemiological information (age groups, onset dates, location). We used the R package ‘‘*o2geosocial*” (v1.0.2)^26^, developed to infer who-infected-whom when genetic sequences are unavailable or uninformative. The package uses the age, location, type of influenza and onset date of each case, the distributions of influenza incubation period and generation time, and estimates of social contact patterns between age groups. It applies Bayesian inference using a Markov-chain Monte Carlo method to jointly estimate the probabilistic transmission trees, and the importation status of each case in each region for each influenza season. The method implemented in the package generates transmission tree samples from the posterior distribution, with a likelihood function relying on various components.

#### Influenza type component

Influenza cases were either diagnosed clinically or by RDTs. RDTs can identify the type of influenza for a given nasopharyngeal swab sample as A, B, or both. We categorized cases in the dataset into Influenza A, B and ‘not attributed’ (clinically diagnosed and A + B coinfected cases) classes. In our model, we assumed that there can be a single type of influenza for each transmission tree. For each influenza (A or B) case, only cases from trees containing the same influenza type could be listed as potential infector, while cases coinfected with both types and clinically diagnosed (less than 1% of all cases) could belong to any tree. This reduced the pool of potential infectors for genotyped cases, and helped the model identify plausible transmission links.

#### Age mixing component

We used a social contact survey study conducted in Japan^27^ to estimate the probability of social contact between age groups. This contact survey was conducted in 2014 and included 1476 households covering all 47 prefectures in Japan. Contacts were grouped into 5-years age groups to derive the social contact matrix. We calculated the weighted average between weekday and weekend contact rates with 5:2 weights. The same matrix was used for all the seasons investigated and the impact of long school holidays on contact patterns was not considered.

#### Conditional reporting ratio

Conditional report ratio refers to the likelihood of missing generation between two cases. It differs from the overall report ratio in that it describes the reporting ratio between reported cases (i.e., it does not consider entirely unreported transmission chains). Influenza surveillance data in Japan is assumed to be high given the cultures of patients-initiated diagnosis during the influenza season^21^. However, in this study, conditional report ratio was estimated using a beta distribution prior (mean 0.9, Standard Deviation: 0.08) as one of the parameters of the model.

#### Time component

For each case, the probabilistic distribution of infection date of each case was inferred from the reported date of onset and distribution of the incubation period. The probability of a case being infected by another case is estimated from generation time of influenza and the number of generations between the two cases. The distributions (shifted Weibull distribution) of incubation period (Mean: 1.63 days, SD: 0.26 days) and generation time (Mean: 3.2 days, SD:0.4 days) of influenza were taken from previous studies^28,29^. Given the distributions of the generation time and incubation period, we assumed that only cases reported less than 8 days prior to the report date of a case could be potential infectors for computational efficiency (Temporal pre-clustering, δ).

#### Spatial component

The estimates of the probability of transmission between regions were calculated using Stouffer’s rank model^30,31^. In the Stouffer’s rank method, rather than the absolute distance between the districts, the connectivity between the districts was estimated as the summed population of all the districts closer to district of infected case than the district of infector case. Stouffer’s distance is calculated as ${p}_{kl}= {m}_{l}^{c}*{(\frac{{m}_{k}}{\sum {i\in\Omega }_{kl} {m}_{i}})}^{a}$ , where m is the population size of the regions, and the regions are defined as

$${\Omega }_{k,l}=\{i:0\le d(i,l)\le d(k,l)\}$$

d (i,*l*) , d(k,*l*) ; distance between regions (i,l) and (k,l).

Then the probability of a case from region *l* infected by a case from region *k* is

$$s(kl)= \frac{{P}_{kl}}{{\sum }_{h}{P}_{hl}}=\frac{{\left(\frac{{m}_{k}}{\sum {i\in\Omega }_{kl} {m}_{i}}\right)}^{a}}{\sum h {\left(\frac{{m}_{k}}{\sum {i\in\Omega }_{kl} {m}_{i}}\right)}^{a}}$$

The parameter $a$ was estimated by the model. We chose to implement a Stouffer’s rank model rather than use a gravity model (which would depend on the absolute number of inhabitants per region and distance) because of the variance in the number of inhabitants per district on Kamigoto Island (from 40 to 3000 inhabitants). This variance would lead to populated areas causing an abnormally high number of transmissions in a gravity model, and underestimate within-region transmission in low populated areas. We assumed that only cases reported in a 100 km radius of a given case could be listed as potential infectors of this case for computational efficiency.

Therefore, the overall log-likelihood that the case *i* was infected by case *j*, belonging to the transmission tree ${\tau }_{j}$ is estimated as^27^

$${L}_{ji} \left({t}_{i},{t}_{j}, \theta \right)= log\left(p\left({\kappa }_{ij}|\Pi \right)*{w}^{\left(\kappa ji\right)} \left({t}_{i}-{t}_{j}\right)* {\alpha }^{\left(\kappa ji\right)} \left({\alpha }_{i}, {\alpha }_{j}\right)*G \left({g}_{i}, {g}_{{\tau }_{j}}\right)*{s}^{\left(\kappa ji\right)} \left({r}_{i}{r}_{j}|a\right)*f({t}_{i}- {T}_{i})\right)$$

where, *k*_*ij*_ is the number of generations between cases $i$ and $j$, equal to 1 if $j$ infected $i$, equal to 2 if there is an unreported case between $j$ and $i$. $G \left({g}_{i}, {g}_{{\tau }_{j}}\right)$ is the likelihood associated with the influenza type (equal to 0 if ${g}_{i}!={g}_{{\tau }_{j}}$, 1 otherwise, and where ${g}_{i}$ is the influenza type reported for $i$, while ${g}_{{\tau }_{j}}$ is the influenza type reported in the transmission tree ${\tau }_{j}$ containing $j$),${\alpha }^{\left(\kappa ji\right)} \left({\alpha }_{i}, {\alpha }_{j}\right)$ is the age component (with ${\alpha }_{i}$ and ${\alpha }_{j}$ the age groups of cases $i$ and $j$),${s}^{\left(\kappa ji\right)} \left({r}_{i}{r}_{j}|a\right)$ is the spatial component (with ${r}_{i}$ and ${r}_{j}$ the regions of residence of $i$ and $j$, and $a$ the parameter of the Stouffer’s rank spatial kernel),$p\left({\kappa }_{ij}|\Pi \right)$ is the likelihood of the conditional report ratio (with $\Pi$ the conditional report ratio of the tree).$w^{{\left( {\kappa ji} \right)}} \left( {t_{i} - t_{j} } \right)*f\left( {t_{i} - T_{i} } \right)$ is the time component (with ${t}_{i}$ and ${t}_{j}$ the estimated infection dates of $i$ and $j$, ${T}_{i}$ the reported onset date of $i$, $w$ the distribution of the generation time, and $f$ the distribution of the incubation period).

Cases identified by the surveillance data as importations and cases with no plausible infectors (i.e., no other case reported within the temporal pre-clustering threshold $\delta$ (8 days), and with the spatial pre-clustering threshold $\gamma$ (100 km radius)) were set as importations in the model. In addition, we used the importation threshold (*λ*) as defined in the package o2geosocial to infer the importation status of cases that were not previously identified as importations. In o2geosocial, for each case and iteration, connections with a likelihood below the importation threshold are defined as non-plausible and removed.

**Figure 2:**
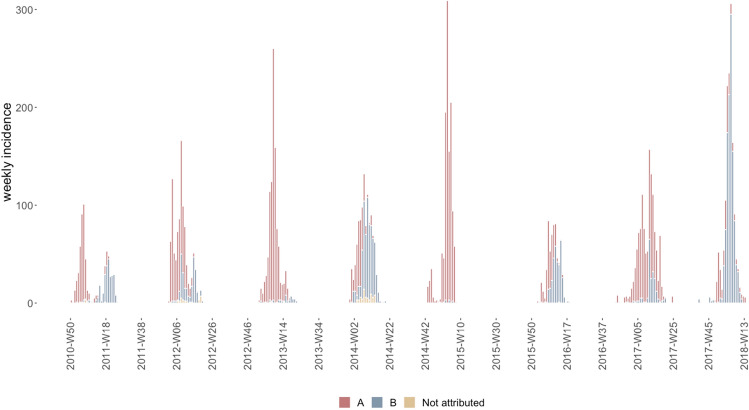
Weekly incidence of RDT-confirmed influenza cases in Kamigoto Island, Japan 2010/11–2017/18 influenza seasons.

The prior distributions used are listed in Table 1. We ran a Monte Carlo Markov Chain (MCMC) with 40,000 iterations to sample from the probabilistic distribution of likely transmission trees. The first 5000 samples were discarded as burn-in and 1 in 50 samples were kept thinning the chain, leaving 700 sampled trees describing the posterior distribution (Supplementary S-Fig. 1). To assess the convergence of the MCMC chain, we run the chain with different initial values and observe their convergence to the same mean and variance for each parameter. The final convergence agreement is shown in the S-Fig. 1. The posterior distribution of the parameters was consistent throughout the chain. The reconstructed cluster size distribution in the inferred trees is shown in supplementary S-Fig. 2. The maps describing the spatial distribution of transmission and importation risks were plotted at the post-code level using the shapefile of the Kamigoto Island obtained from ESRI Japan’s website^22^.

**Table 1:** Parameters used in the Bayesian analysis.

Parameters	Symbols	Values	Ref/comments
Incubation period	f (t)	Shifted Weibull distribution with: Mean: 1.63 days SD: 0.26 days	(28)
Generation time	w (t)	Shifted Weibull distribution with: Mean: 3.2 days SD: 0.4 days	(29)
Conditional report ratio	$\Pi$	Prior: beta distribution, mean = 0.9, s.d. = 0.08	Estimated
Spatial parameter	a	Prior: uniform distributions between 0 and 5	Estimated
Spatial pre-clustering	*γ*	100 km	Fixed
Temporal pre-clustering	*δ*	8 days (based on incubation period and generation times)	Fixed
Importation threshold (absolute) 5 × log0.05 = − 15	*λ*	Default in o2geosocial package	Fixed

### Regression analysis to identify factors associated with onwards transmission

The inferred probabilistic transmission trees were then used to identify factors consistently associated with increased risks of onwards transmission. To do so, we implemented a negative binomial regression analysis on each sampled transmission tree kept from the MCMC and pooled the results together. The outcome variable of the analysis was the number of onwards transmission per case. The sociodemographic variables used as explanatory variables were: (1) age group, (2) vaccination status, (3) vaccine coverage in the district of the reported case, (4) type of flu (5) number of inhabitants in the district of the reported case, (6) mean household size, (7) average vaccination coverage in neighboring districts, (8) epidemic year, and (9) within-year seasonality (sin and co-sin).

We grouped the age of cases into categories: < 3, 4–6, 7–12, 13–18, 19–64, 65–74 and ≥ 75 years-old. Individual vaccination status was categorized as vaccinated, unvaccinated, and unknown. We controlled for the impact of seasonality (the peak of the influenza in each influenza season) on transmission by adding two covariates (sine–cosine) in the regression analysis. The vaccination coverage in nearby district was defined as the population- and district-wise average coverage in the neighbors of each region.

The regression was run independently on each transmission network sampled from the MCMC (i.e., 700 samples, obtained after removing the burn-in phase and thinning the chain). The coefficients were then pooled together following “Rubin’s rules” to identify factors consistently associated with increased transmission risks across all samples^32^. We examined collinearity among the variables before running the regression models. We run different models to evaluate the robustness of our results: in model II, we categorized the population per district and vaccine coverage variables, and in model III, we also removed the seasonality variables.

All analyses were performed with R software, version 4.1.2 (R Foundation for Statistical Computing)^33^.

### Ethics approval

This study is a secondary analysis of data collected as part of routine surveillance of influenza in Japan. All methods were performed in accordance with the relevant guidelines and regulations. The research was approved by the institutional review boards of Kamigoto Hospital, Nagasaki University Research Ethics Committee (reference number 200619236), and the London School of Hygiene and Tropical Medicine Research Ethics Committee (reference number 26706). Both Nagasaki University and London School of Hygiene and Tropical Medicine granted waivers for obtaining informed consent due to the nature of this retrospective study and the preserved anonymity of patients.

### Role of the funding source

SMH was supported by WISE Program (Doctoral Program for World-leading Innovative & Smart Education) of Ministry of Education, Culture, Sports, Science and Technology. AR was supported by the National Institute for Health Research (NIHR200908). The funder of the study had no role in the study design, data collection, data analysis, data interpretation, or the writing of the report. The corresponding author had full access to all the data in the study and had final responsibility for the decision to submit for publication.

## Results

### Epidemiological analysis

In Kamigoto Island, a total of 8497 RDT-confirmed influenza cases were reported from 2010/11 to 2017/18. Figure 2 shows the distribution of influenza cases by RDT-confirmed influenza type. A-H3N2 subtype dominated in majority of the seasons, while influenza B subtype dominated in 2017/18. (Fig. 2, supplementary S-Table 1).

**Figure 3:**
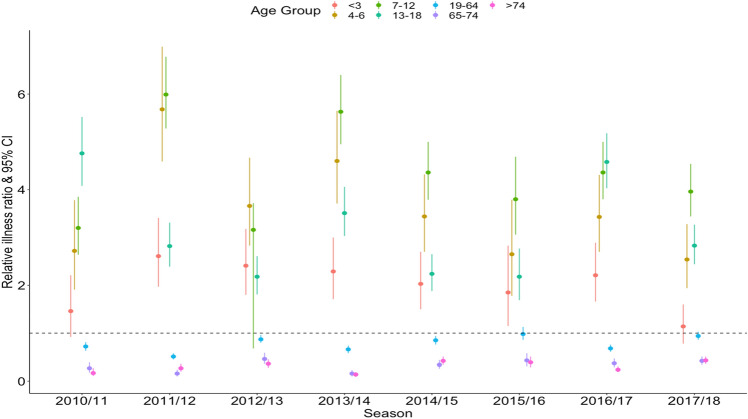
Relative illness ratio of influenza.

To assess which age groups were more likely to be infected, we calculated the relative illness ratio (RIR) of each age group in each epidemic season (Fig. 3). Consistently across all epidemics, RIRs were higher in the pre-school and school children age-group (below 19 years old). The RIR was lower in other age groups in all epidemic seasons. The attack rate was highest among school-aged children (4–6, 7–12 and 13–18) years old across all the epidemic seasons (Supplementary S-Table 2).

### Reconstruction of probabilistic transmission trees

**Figure 4:**
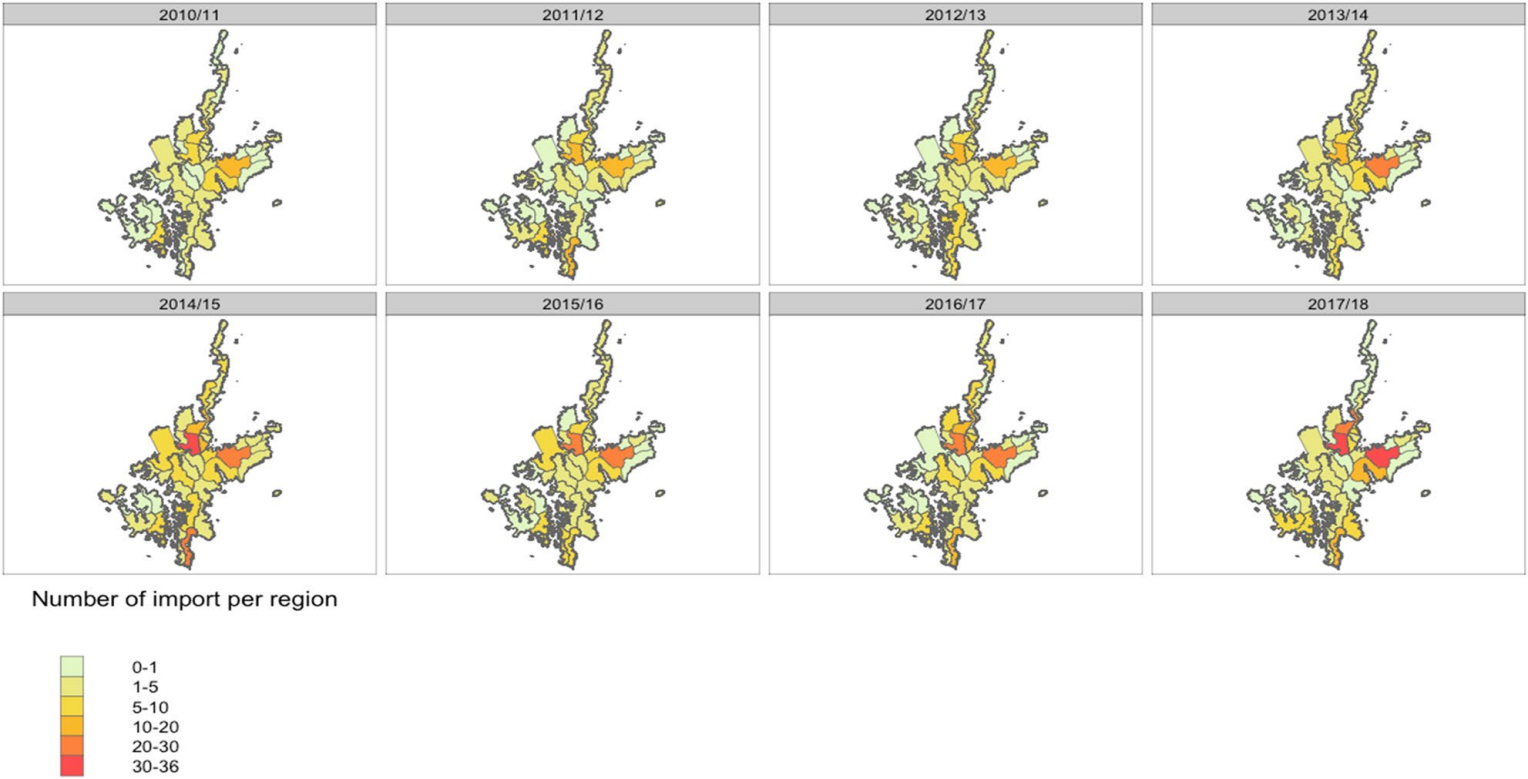
Median number of imported cases stratified by district and by season (2010/11–2017/18).

We used the R package o2geosocial to infer who-infected-whom among cases reported between 2010 to 2018 and estimate the number of secondary transmissions per case. The conditional report ratio ranged between 86 and 91% (Supplementary S-Fig. 1). The inference method therefore considered that most of the reported transmission chains captured by the surveillance data were complete, with a low risk of missed generation between reported cases. The spatial parameter ranged between 1.6 and 1.7 (Supplementary S-Fig. 1), which corresponds to a decrease in the probability of connection between regions as the number of inhabitants living in the area between these two regions increases. Figure 4 shows the inferred distribution of imported cases in each district for each season. There were 1050 cases classified as importations in the inferred transmission trees over the study period. MCMC inference therefore identified an additional 91 imported cases which were not classified as imports in the epidemiological data. These 91 cases were cases with no plausible infector and highlight the good performance of the surveillance system. The regions associated with most importations were consistent across epidemic seasons. Three districts (Tainoura-go, Arikawa-go, and Aokata-go) were estimated to have recorded most imported cases (orange and red) across the seasons. Tainoura-go and Arikawa-go are the main port areas of the island and are therefore the main connection from and to areas outside the Kamigoto island. Aogata-go is a more urban area of the town and the district and contains Kamigoto hospital, the only hospital within the island. Arikawa-go and Aogata-go districts are the tourist attractions within the island. The district on the southern part of the island (Narao-go), another port area, was also suggested to have large number of imports in alternate years examined. We also found a gradual increase in the overall number of imported cases over the years studied (73 import cases in 2010/11 to 201 import cases in 2017/18).

**Figure 5:**
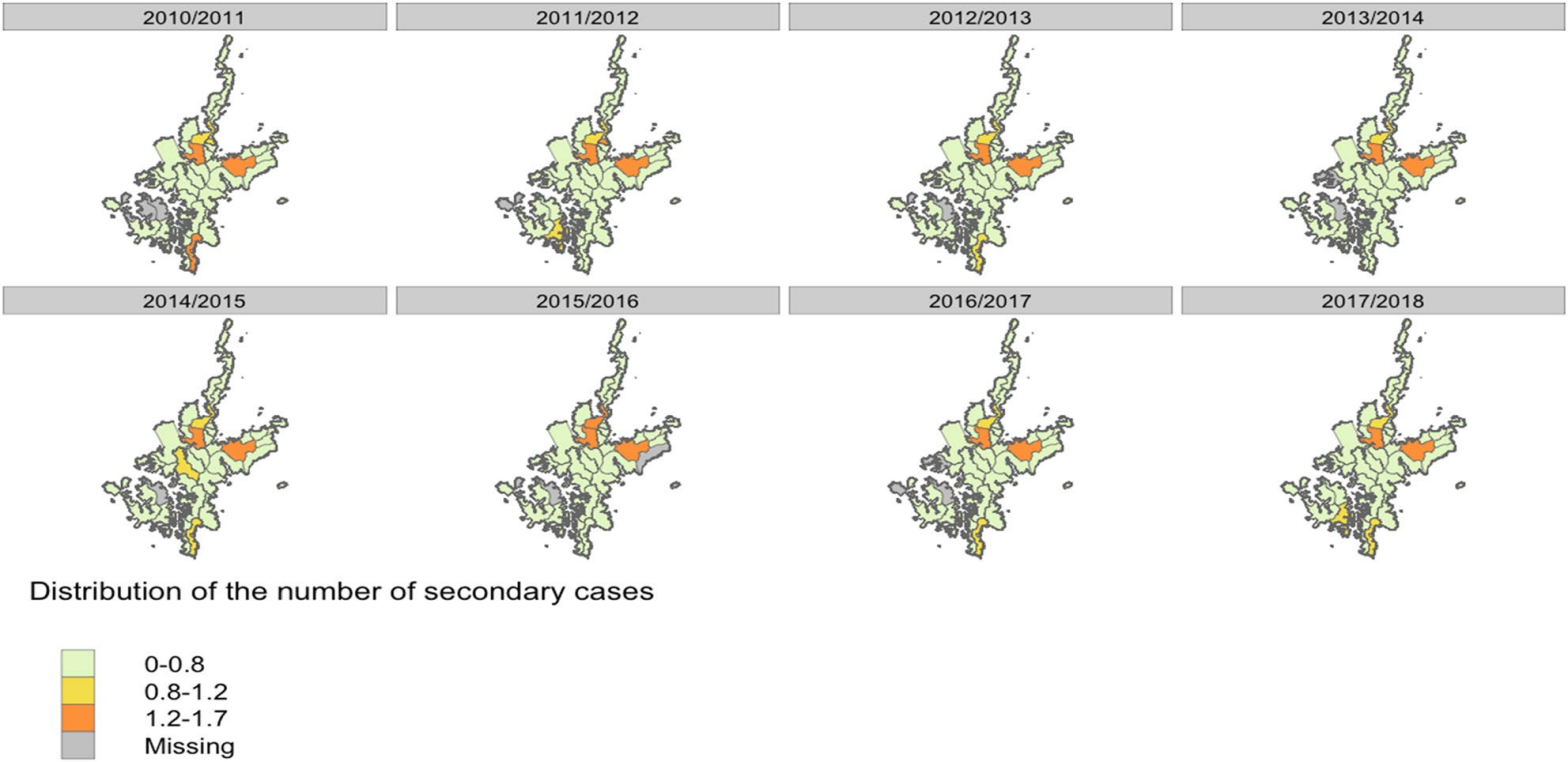
Median number of secondary cases per each influenza case, stratified by district and by season (2010/11–2017/18).

We then used the set of inferred transmission trees to compute the average number of secondary cases per primary case or individual reproduction number (R_eff_) in each district. The geographical distribution of individual reproduction number (R_eff_) was very similar across the epidemic seasons (Fig. 5). The districts at higher risks of importations also consistently had the highest individual reproduction number (R_eff_ ≥ 1.2) (Fig. 5, Supplementary S-Fig. 4). The R_eff_ was below 0.8 in the majority of the remaining districts, with localized outbreaks causing increases in the number of secondary cases per case in certain years (e.g., in the south of the island in 2014/15 and 2017/18) (Fig. 5, supplementary S-Fig. 3). We categorized the average number of secondary cases for each case, as: (1) no onward transmission, (2) moderate transmitters (1–3 cases), or (3) high transmitters (more than 3 cases). Preschool (< 3 years old), adult (19–64) and older age group (65–74, > 74 years old) had lower onward transmission while the school age group (4–6, 7–12 and 13–18) were more likely to have higher number of secondary cases generated (supplementary S-Fig. 5 and S-Fig. 6). The probability of transmission between and within district are displayed in supplementary S-Fig. 7.

### Determinants of onwards transmission

Next, we identified the factors consistently associated with increases in the risk of onwards transmission across the inferred transmission trees. In each transmission tree from the posterior distribution (i.e., 700 samples), we computed the number of secondary transmissions per case, and ran a negative binomial regression analysis to explore characteristics associated with the risk of onwards transmission. The coefficients were then pooled across all regressions.

We found that age, number of inhabitants per district, district- level vaccination coverage, type of influenza and epidemic season had significant associations with the number of secondary cases per case (Table 2). Pre-school and school-aged children (aged 4–18) were significantly associated with higher secondary cases (incidence rate ratio (IRR) = 1.38 (95% confidence interval (CI) 1.21, 1.57), 1.45 (95% CI 1.33, 1.59), and 1.51 (95% CI 1.37, 1.66) compared to the adult (aged 19–64) group. On the contrary, adults over 65 years were associated with significantly fewer secondary cases (IRR = 0.72 (95% CI 0.58, 0.89) in aged 65–74 and 0.76 (95% CI 0.63, 0.91) in aged 75 years and over).

**Table 2:** Association factors to the number of secondary cases generated, Kamigoto island, Japan, 2010/11–2017/18.

Variable	Secondary cases	IRR	95% CI	p-value
Intercept	0.01		(0.01, 0.03)	< 0.001**
Age group, years				
< 3		0.95	(0.78, 1.14)	0.574
4–6		1.38	(1.21, 1.57)	< 0.001**
7–12		1.45	(1.33, 1.59)	< 0.001**
13–18		1.51	(1.37, 1.66)	< 0.001**
19–64		1	Reference	
65–74		0.72	(0.58, 0.89)	0.002*
≥ 75		0.76	(0.63, 0.91)	0.003*
RDT results				
Flu A		1	Reference	
Flu B		0.83	(0.77, 0.90)	< 0.001**
Not attributed		0.68	(0.48, 0.98)	0.037*
Vaccination history				
No		1	Reference	
Yes		1.06	(0.96, 1.17)	0.278
Unknown		1.10	(0.95, 1.27)	0.213
Mean household size		1.04	(0.66, 1.62)	0.873
Population per district		2.00	(1.89, 2.12)	< 0.001**
Proportion unvaccinated in the district		1.45	(1.02, 2.05)	0.037*
Average proportion unvaccinated in neighboring districts		2.41	(1.11, 5.21)	0.026*
Influenza season				
2010/11		1	Reference	
2011/12		0.87	(0.76, 1.00)	0.044
2012/13		0.96	(0.84, 1.10)	0.580
2013/14		0.95	(0.84, 1.09)	0.476
2014/15		0.90	(0.79, 1.03)	0.141
2015/16		1.28	(1.06, 1.54)	0.010*
2016/17		0.91	(0.79, 1.06)	0.232
2017/18		1.31	(1.10, 1.57)	0.003*
Seasonality				
Sin		0.99	(0.95, 1.04)	0.769
Cosin		1.01	(0.96, 1.05)	0.823

Note: *<0.05; **<0.001.

Cases living in districts that achieved lower vaccine coverage were more likely to cause further transmission (IRR = 1.45 (95% CI 1.02, 2.05)). There was a significant positive association between the number of inhabitants per district and the number of onwards transmissions (IRR = 2.00 (95% CI 1.89, 2.12)). The average proportion of unvaccinated individuals in neighboring regions was also associated with an increased risk of secondary transmissions 2.41 (95% CI 1.11, 5.21). Type B influenza were less likely to generate more secondary transmission compared to type A influenza (IRR = 0.83 (95% CI 0.77, 0.90)). Epidemic seasons 2015/16 and 2017/18 were associated with higher transmission compared to 2010/11 (IRR = 1.28 (95% CI 1.06, 1.54), 1.31 (95% CI 1.01, 0.57) respectively). There was no significant association between individual vaccination status, mean household size, seasonality and onwards transmission. The results were consistent with different models. (Supplementary S-Table 3).

## Discussion

We analysed the dynamics of influenza transmission in a remote island setting in Japan (Kamigoto Island), using fine scale influenza surveillance data collected between 2010 and 2018. Kamigoto island provides a unique opportunity to explore the dynamics of influenza transmission in an heterogenous population, with limited mobility*.* We analysed the age-specific influenza burden, the importation status of the cases, and the risk of secondary transmission for each case by reconstructing transmission trees using a Bayesian approach. We also explored factors associated with onwards influenza transmission.

The result from our study adds to the evidence that children play an important role in influenza outbreaks^34–42^. Across all epidemic seasons, pre-school, and school-aged children (i.e., 4–18-year-old) were infected at higher rate than the other age groups (Fig. 3). Previous studies have reported that age distribution patterns vary between the influenza subtypes/lineages, with school-aged children most likely to be affected by A(H3N2) and B^15,36^. In Japan A(H3N2) was the dominant subtypes in four of the eight seasons, A/H1N1pmd09 in three seasons (2010/11, 2013/14 and 2015/2016) whereas B was predominant 2017/18 (supplementary S-Table 1). We also found that school-aged children were associated with the highest number of secondary cases generated. This is in line with studies reporting that social contact rates within the same age group is higher among school-aged children^27,34^. On the other hand, we found that adults over 65 years-old were associated with the lowest influenza attack rate, and lower risks of onwards transmission. Similar results were previously reported in a study conducted in Italy^15^.

Our study highlighted that vaccination had different impacts on influenza dynamics. Firstly, high vaccine coverage in a district was consistently associated with lower levels of transmission, highlighting the importance of vaccination campaigns to reduce the risks of outbreaks through direct and indirect protection. Moreover, high vaccine coverage in regions neighboring a given district were also associated with reduced transmission risks. This may show the importance of reaching homogeneously high vaccine coverage, rather than focusing on a limited number of districts, to reduce opportunities for onward transmission. However, the spatial heterogeneity of the median number of secondary cases per region shows that active transmission clusters was only observer around a limited number of districts (Fig. 5). More analysis, potentially using mechanistic transmission models, is needed to compare the impact of different vaccination strategies.

In this study, the vaccine status of the individual cases was not associated with changes in the risk of secondary cases, suggesting that while vaccination is protective against infection, it might not necessarily prevent onwards transmission if breakthrough infection happens. A study conducted among children in Matsumoto city, Japan also reported an association between vaccination and reduction in susceptibility, with vaccination having a more limited association with onwards transmission^43^. Recently published studied also reported the low degree of indirect protection by childhood vaccination supporting the results of the current study^44^.

The RIR and attack rate was highest among school-aged children despite very high childhood vaccination coverage in the island (Supplementary S-Table 4). RIR and attack rate was lowest among the elderly age group (> 65 years old), and the vaccination coverage among these age group were above 70% in almost all epidemic seasons (Supplementary S-Table 2 and S-Table 4). The highest attack rates among children implies that their higher number of contacts was not fully offset by the high immunization rates. The majority of the reported cases were vaccinated, which indicates that although it does provide protection, the efficacy of the vaccine is not perfect. Previous studies on vaccine effectiveness reported the negative impact of repeated vaccination in children without prior history of natural infection^45–48^. The type of influenza vaccine may also play a role, since Japan uses the inactivated influenza vaccine while other countries (for example: UK)^42,49^ use live attenuated influenza vaccine (LAIV). Similar results were previously reported when comparing the incidence among age groups with vaccination status in the United States: estimated incidence for children below 18 years old was 8.7% while it was 5.1% for adults after adjustment for median vaccine coverage and effectiveness^50^.

To summarise, our study reveals different effects of vaccination on influenza transmission in Kamigoto Island: Firstly, although the proportion of vaccinated cases in our dataset implies that the protection provided by vaccines may not be perfect, cases reported in highly vaccinated regions were associated with lower risk of secondary transmission. This suggests that the protection provided by high vaccine coverage in a region did minimize opportunities for onward transmission (by reducing the pool of susceptible individuals available to be infected). Vaccination history of cases (i.e. conditioned that they were infected) were not associated with lower risk of onward transmission, showing that although the vaccine may provide protection against infection, we cannot conclude that it provided significant protection against onward transmission from those who experienced a ‘breakthrough’ infection.

The inferred transmission trees suggested that the distribution of influenza transmission within the island was similar across the epidemic seasons with the same districts having the highest number of importation and secondary transmissions per case (Arikawa-go, Tainoura-go, Aogata-go and Narao-go). With the presence of the ports connecting the islands to mainland Japan, major tourists’ attraction sides of the island, and the highest number of inhabitants, these districts provide the best opportunities for the influenza transmission in the community. Moreover, people residing within the island but working outside (e.g., Nagasaki city) need to take a ferry from these ports to commute daily to their workplaces. This connectivity to the outside of the island likely contributes to the importation of influenza into the island.

The determinants of onward transmission identified by the regression analysis were consistent throughout all samples generated in the transmission tree inference. The Bayesian inference model, implemented using o2geosocial, was able to identify a robust history of transmission from routinely collected surveillance data (location, age, timing of disease onset). The different parameters estimated in the MCMC converged to consistent means and variances. The sampled transmission tree could not be compared to sequence data or contact tracing investigations to assess their accuracy, however, the results and estimates from the inference are in line with prior results, for instance on the high number of infectious contacts among children^27^, or the low risk of unreported cases in Japan^39^, and were robust to sensitivity analysis. We believe this indicates that our findings are reliable. This analysis is therefore an example of how even limited surveillance data can be used to reconstruct complex dynamics of transmission, highlight factors associated with transmission, and inform how to limit the risks of flu outbreak.

There are several limitations to our study. First, we assumed the same reporting rates across all the seasons we analyzed. Second, influenza transmission is suggested to be influenced by some meteorological variables such as temperature, absolute humidity and precipitation^16,51^. We did not consider these factors in our analysis. Third, we did not account for the effect of nonpharmaceutical interventions, such as mask wearing and hand hygiene. The period of school closure can also have impact on the transmission dynamics; however, this was not included this in our study. Lastly, district with higher proportion of children may influence the onwards transmissibility and higher infection rates. However, we do not have access to the distribution of age group by district.

## Conclusion

Our study used geospatial method to better describe spatiotemporal patterns in a fine-scale epidemiological setting. We show that age, population density, local vaccination coverage, and dominant strain of influenza play a significant role in shaping recurrent influenza spatial patterns across the seasons studied. The local vaccine coverage was found to be strongly associated with the risk of onwards transmission, highlighting the importance to focus public health efforts on achieving high vaccine coverage throughout the island or prioritizing vaccination in areas with high transmission rates. The individual vaccine status may not prevent onwards transmission, though it reduces the susceptibility to infection. Thus, the other social precaution measures such as social distancing, handwashing, and wearing a mask must be practiced once someone is infected. Similar analysis could potentially be reproduced in similar settings (e.g. Oceania and pacific island countries), to study the transmission of influenza or other infectious disease such as COVID-19.

## Supplementary Information

Supplementary Information.

## Data Availability

All data generated or analysed during this study, is publicly available on Github repository (https://github.com/Su06690/Temporal-Spatial-Flu-spread-Island-community).

## References

[1] Gordon A, Reingold A (2018). The burden of influenza: A complex problem. Curr. Epidemiol. Rep..

[2] Troeger CE, Blacker BF, Khalil IA (2019). Mortality, morbidity, and hospitalisations due to influenza lower respiratory tract infections, 2017: An analysis for the Global Burden of Disease Study 2017. Lancet Respir. Med..

[3] Alonso WJ, Viboud C, Simonsen L, Hirano EW, Daufenbach LZ, Miller MA (2007). Seasonality of influenza in Brazil: A traveling wave from the Amazon to the subtropics. Am. J. Epidemiol..

[4] Stark JH, Sharma R, Ostroff S (2012). Local spatial and temporal processes of influenza in Pennsylvania, USA: 2003–2009. PLoS ONE.

[5] Viboud C, Bjornstad ON, Smith DL, Simonsen L, Miller MA, Grenfell BT (2006). Synchrony, waves, and spatial hierarchies in the spread of influenza. Science.

[6] Paget J, Marquet R, Meijer A, van der Velden K (2007). Influenza activity in Europe during eight seasons (1999–2007): An evaluation of the indicators used to measure activity and an assessment of the timing, length and course of peak activity (spread) across Europe. BMC Infect. Dis..

[7] Viboud C, Boelle PY, Pakdaman K, Carrat F, Valleron AJ, Flahault A (2004). Influenza epidemics in the United States, France, and Australia, 1972–1997. Emerg. Infect. Dis..

[8] Bonabeau E, Toubiana L, Flahault A (1998). The geographical spread of influenza. Proc. Biol. Sci..

[9] Mugglin AS, Cressie N, Gemmell I (2002). Hierarchical statistical modelling of influenza epidemic dynamics in space and time. Stat. Med..

[10] Sakai T, Suzuki H, Sasaki A, Saito R, Tanabe N, Taniguchi K (2004). Geographic and temporal trends in influenzalike illness, Japan, 1992–1999. Emerg. Infect. Dis..

[11] Ye C, Zhu W, Yu J (2019). Understanding the complex seasonality of seasonal influenza A and B virus transmission: Evidence from six years of surveillance data in Shanghai, China. Int. J. Infect. Dis..

[12] Charu V, Zeger S, Gog J (2017). Human mobility and the spatial transmission of influenza in the United States. PLoS Comput. Biol..

[13] Virk RK, Gunalan V, Lee HK (2017). Molecular evidence of transmission of influenza A/H1N1 2009 on a university campus. PLoS ONE.

[14] Dalziel BD, Kissler S, Gog JR (2018). Urbanization and humidity shape the intensity of influenza epidemics in U.S. cities. Science.

[15] Trentini F, Pariani E, Bella A (2022). Characterizing the transmission patterns of seasonal influenza in Italy: Lessons from the last decade. BMC Public Health.

[16] Huang X, Mengersen K, Milinovich G, Hu W (2017). Effect of weather variability on seasonal influenza among different age groups in Queensland, Australia: A Bayesian spatiotemporal analysis. J. Infect. Dis..

[17] Lee JS, Kawakubo K (2003). Influenza vaccination coverage in Japan. Lancet.

[18] Health Service Bureau Ministry of Health Labour and Welfare. The guidelines for National Epidmeiological Surveillance of Infectious Diseases: Influenza Health Service Bureau Ministry of Health Labour and Welfare.: Health Service Bureau Ministry of Health Labour and Welfare. p. Japanese version.

[19] Yamazaki M, Mitamura K, Ichikawa M (2004). Evaluation of flow-through immunoassay for rapid detection of influenza A and B viruses. Kansenshogaku Zasshi.

[20] Hara M, Sadamasu K, Takao S (2006). Evaluation of immunochromatography test for rapid detection of influenza A and B viruses using real-time PCR. Kansenshogaku Zasshi.

[21] Tsuzuki S, Yoshihara K (2020). The characteristics of influenza-like illness management in Japan. BMC Public Health.

[22] Japan E. National municipal boundary data. ESRI Japan ESRI Japan (2011).

[23] Lemaitre M, Carrat F (2010). Comparative age distribution of influenza morbidity and mortality during seasonal influenza epidemics and the 2009 H1N1 pandemic. BMC Infect. Dis..

[24] Minodier L, Arena C, Heuze G (2014). Epidemiology and viral etiology of the influenza-like illness in corsica during the 2012–2013 Winter: an analysis of several sentinel surveillance systems. PLoS ONE.

[25] Karageorgopoulos DE, Vouloumanou EK, Korbila IP, Kapaskelis A, Falagas ME (2011). Age distribution of cases of 2009 (H1N1) pandemic influenza in comparison with seasonal influenza. PLoS ONE.

[26] Robert A, Funk, S. & Kucharski, A. J. o2geosocial: Reconstructing who-infected-whom from routinely collected surveillance data [version 2; peer review: 1 approved, 2 approved with reservations]. F1000Research **10**, 31 (2021). 10.12688/f1000research.28073.1 PMC10044721 36998981

[27] Munasinghe L, Asai Y, Nishiura H (2019). Quantifying heterogeneous contact patterns in Japan: a social contact survey. Theor. Biol. Med. Model..

[28] Cori A, Valleron AJ, Carrat F, Scalia Tomba G, Thomas G, Boelle PY (2012). Estimating influenza latency and infectious period durations using viral excretion data. Epidemics.

[29] Petrie JG, Ohmit SE, Cowling BJ (2013). Influenza transmission in a cohort of households with children: 2010–2011. PLoS ONE.

[30] Stouffer SA (1940). Intervening opportunities: A theory relating mobility and distance. Am. Sociol. Rev..

[31] Noulas A, Scellato S, Lambiotte R, Pontil M, Mascolo C (2012). A tale of many cities: Universal patterns in human urban mobility. PLoS ONE.

[32] Rubin DB (1987). Multiple Imputation for Nonresponse in Surveys.

[33] R Development Core Team (2008). R: A Language and Environment for Statistical Computing.

[34] Muller NF, Wuthrich D, Goldman N (2020). Characterising the epidemic spread of influenza A/H3N2 within a city through phylogenetics. PLoS Pathog..

[35] Reed C, Katz JM, Hancock K, Balish A, Fry AM (2012). Prevalence of seropositivity to pandemic influenza A/H1N1 virus in the United States following the 2009 pandemic. PLoS ONE.

[36] Turbelin C, Souty C, Pelat C (2013). Age distribution of influenza like illness cases during post-pandemic A(H3N2): Comparison with the twelve previous seasons, in France. PLoS ONE.

[37] Cauchemez S, Valleron AJ, Boelle PY, Flahault A, Ferguson NM (2008). Estimating the impact of school closure on influenza transmission from Sentinel data. Nature.

[38] Oliveira CR, Costa GSR, Paploski IAD (2016). Influenza-like illness in an urban community of Salvador, Brazil: Incidence, seasonality and risk factors. BMC Infect. Dis..

[39] Endo A, Uchida M, Kucharski AJ, Funk S (2019). Fine-scale family structure shapes influenza transmission risk in households: Insights from primary schools in Matsumoto city, 2014/15. PLoS Comput. Biol..

[40] Zhou L, Yang H, Kuang Y (2019). Temporal patterns of influenza A subtypes and B lineages across age in a subtropical city, during pre-pandemic, pandemic, and post-pandemic seasons. BMC Infect. Dis..

[41] Caini S, Spreeuwenberg P, Kusznierz GF (2018). Distribution of influenza virus types by age using case-based global surveillance data from twenty-nine countries, 1999–2014. BMC Infect. Dis..

[42] Baguelin M, Flasche S, Camacho A, Demiris N, Miller E, Edmunds WJ (2013). Assessing optimal target populations for influenza vaccination programmes: An evidence synthesis and modelling study. PLoS Med..

[43] Endo A, Uchida M, Hayashi N (2021). Within and between classroom transmission patterns of seasonal influenza among primary school students in Matsumoto city, Japan. Proc. Natl. Acad. Sci..

[44] Tsang TK, Fang VJ, Ip DKM (2019). Indirect protection from vaccinating children against influenza in households. Nat. Commun..

[45] Saito N, Komori K, Suzuki M, Kishikawa T, Yasaka T, Ariyoshi K (2018). Dose-dependent negative effects of prior multiple vaccinations against influenza A and influenza B among schoolchildren: A study of Kamigoto Island in Japan during the 2011–2012, 2012–2013, and 2013–2014 influenza seasons. Clin. Infect. Dis..

[46] Saito N, Komori K, Suzuki M (2017). Negative impact of prior influenza vaccination on current influenza vaccination among people infected and not infected in prior season: A test-negative case-control study in Japan. Vaccine.

[47] Hoskins TW, Davies JR, Smith AJ, Miller CL, Allchin A (1979). Assessment of inactivated influenza-A vaccine after three outbreaks of influenza A at Christ's Hospital. Lancet.

[48] Jones-Gray E, Robinson EJ, Kucharski AJ, Fox A, Sullivan SG (2023). Does repeated influenza vaccination attenuate effectiveness? A systematic review and meta-analysis. Lancet Respir. Med..

[49] Hodgson D, Baguelin M, van Leeuwen E (2017). Effect of mass paediatric influenza vaccination on existing influenza vaccination programmes in England and Wales: a modelling and cost-effectiveness analysis. Lancet Public Health.

[50] Tokars JI, Olsen SJ, Reed C (2018). Seasonal incidence of symptomatic influenza in the United States. Clin. Infect. Dis..

[51] Barreca AI, Shimshack JP (2012). Absolute humidity, temperature, and influenza mortality: 30 years of county-level evidence from the United States. Am. J. Epidemiol..

