# Supplementary material for: Transmission dynamics of seasonal influenza in a remote island population
Source: Sci Rep. 2023 Apr 3;13:5393. doi: 10.1038/s41598-023-32537-0 (PMC10068240; doi:10.1038/s41598-023-32537-0)
Supplement: Supplementary file 1 — Supplementary Information. [file 41598_2023_32537_MOESM1_ESM.pdf]

Supplementary

Transmission dynamics of seasonal influenza in remote island populations: epidemiological and modelling Study

Su Myat Han[1,2*], Alexis Robert[2,3], Shingo Masuda[1, 4], Takahiro Yasaka[4], Satoshi Kanda[4], Kazuhiri Komori[4], Nobuo Saito[5,6], Motoi Suzuki[1,7], Akira Endo[1,2,3], Marc Baguelin[2,8], Koya Ariyoshi[1,6]

1. School of Tropical Medicine and Global Health, Nagasaki University, Nagasaki, Japan
2. Department of Infectious Disease Epidemiology, Faculty of Epidemiology and Population Health, London School of Hygiene and Tropical Medicine, London, United Kingdom
3. Centre for the Mathematical Modelling of Infectious Diseases, London School of Hygiene & Tropical Medicine, Keppel Street, London, UK
4. Department of Internal Medicine, Kamigoto Hospital, Kamigoto, Japan
5. Department of Microbiology, Faculty of Medicine, Oita University, Yufu, Japan
6. Department of Clinical Medicine, Institute of Tropical Medicine, Nagasaki University, Nagasaki, Japan
7. Infectious Disease Surveillance Center, National Institute of Infectious Diseases, Tokyo, Japan
8. MRC Centre for Global Infectious Disease Analysis; and the Abdul Latif Jameel Institute for Disease

S Table 1: Circulating strains of influenza by season (https://www.niid.go.jp/niid/images/idsc/iasr/39/465e.pdf)

	**2010/11**	**2011/12**	**2012/13**	**2013/14**	**2014/15**	**2015/16**	**2016/17**	**2017/18**
**Circulating strains**								
A/H1N1pmd09	52%	0.2%	2%	43%	1%	**49%**	4%	23%
A/H3N2	32%	71%	76%	21%	85%	7%	78%	32%
B	15%	28%	21%	36%	14%	44%	18%	**45%**
**Vaccine strains**	A/California/7/2009 [A(H1N1) pdm09]<br><br>A/Victoria/210/2009 [A(H3N2)]<br><br>B/Brisbane/60/2008 [B(Victoria)]	A/California/7/2009 [A(H1N1) pdm09]<br><br>A/Victoria/210/2009 [A(H3N2)]<br><br>B/Brisbane/60/2008 [B(Victoria)]	A/California/7/2009 [A(H1N1) pdm09]<br><br>A/Victoria/361/2011 [A(H3N2)]<br><br>B/ Wisconsin /01/2010 [B(Yamgata)]	A/California/7/2009 [A(H1N1) pdm09]<br><br>A/Texas/50/2012 [A(H3N2)]<br><br>B/ Massachusetts /02/2012 [B(Yamgata)]	A/California/7/2009 [A(H1N1) pdm09]<br><br>A/New York/39/2012 [A(H3N2)]<br><br>B/ Massachusetts /02/2012 [B(Yamgata)]	#<br>A/Switzerland/9715293/2013 [A(H3N2)]<br><br>B/Phuket/3073/2013 [B(Yamgata)]<br><br>B/Texas/2/2013 [B(Victoria)]	A/California/7/2009 [A(H1N1) pdm09]<br><br>A/Switzerland/9715293/2013 [A(H3N2)]<br><br>B/Phuket/3073/2013 [B(Yamgata)]<br><br>B/Texas/2/2013 [B(Victoria)]	A/Michigan/45/2015-like A/Singapore/GP1908/2015 [A(H1N1) pdm09]<br><br>A/Hong Kong/4801/2014 [A(H3N2)]<br><br>B/Phuket/3073/2013 [B(Yamgata)]<br><br>B/Texas/2/2013 [B(Victoria)]
**Circulating strains**								
A/H1N1pmd09	Vaccine match	Vaccine match (50%)	Vaccine match (97%)	Vaccine match	Vaccine match	Vaccine match	**Vaccine mismatch**	Vaccine match
A/H3N2	Vaccine match	Vaccine match	Vaccine match	Vaccine match	**Vaccine mismatch**	**50-60% match**	**Vaccine mismatch**	**Vaccine match (50-60%)**
B	Vaccine match	Vaccine match	Vaccine match	Vaccine match	Vaccine match	Vaccine match	Vaccine match	Vaccine match

S-Table 2: Incidence attack rate per 1,000 individuals at risk, stratified by age group in Kamigoto island, Japan over (2010/11- 2017/18) influenza season

Season	<3	4-6	7-12	13-18	19-64	65-74	≥75	Total
**2010/11**	42.6	79.4	93.3	138.7	20.9	7.8	5.0	29.2
	(27.0, 63.9)	(56.5, 108.6)	(77.5, 111.3)	(120.0, 159.5)	(18.3, 23.6)	(5.2, 11.4)	(3.1, 7.7)	(27.0, 31.4)
**2011/12**	115.1	250.5	264.0	124.5	22.5	6.8	12.0	44.1
	(88.0, 147.8)	(206.9, 300.7)	(235.9, 294.5)	(106.4, 144.6)	(19.9, 25.4)	(4.3, 10.4)	(8.9, 15.7)	(41.4, 46.9)
**2012/13**	113.1	171.5	148.3	102.2	41.0	21.8	16.7	46.9
	(85.5, 146.9)	(135.3, 214.3)	(126.9, 172.4)	(85.6, 121.2)	(37.4, 44.9)	(16.9, 27.5)	(13.1, 21.1)	(44.1, 49.8)
**2013/14**	115.3	231.5	283.7	176.9	33.2	7.9	6.9	50.4
	(86.9, 150.1)	(188.3, 281.5)	(252.8, 317.4)	(154.1, 202.0)	(29.9, 36.7)	(5.1, 11.7)	(4.6, 9.8)	(47.4, 53.4)
**2014/15**	115.3	195.1	247.2	127.0	48.2	19.5	23.6	56.7
	(85.3, 152.4)	(154.4, 243.1)	(217.4, 280.1)	(107.5, 148.9)	(44.1, 52.5)	(14.9, 25.0)	(19.2, 28.6)	(53.6, 60.0)
**2015/16**	57.9	82.7	118.9	68.1	30.8	13.4	12.1	31.2
	(36.3, 87.7)	(56.2, 117.3)	(97.1, 144.0)	(53.6, 85.4)	(27.4, 34.4)	(9.8, 18.0)	(9.1, 15.9)	(28.9, 33.8)
**2016/17**	133.7	207.3	263.8	276.5	41.0	22.2	14.6	60.4
	(101.0, 173.6)	(164.2, 258.4)	(232.3, 298.3)	(246.6, 309.1)	(37.2, 45.1)	(17.4, 28.0)	(11.3, 18.7)	(57.1, 63.9)
**2017/18**	81.9	183.4	285.4	204.2	68.0	30.2	31.0	72.1
	(56.4, 115.0)	(140.6, 235.2)	(250.6, 323.7)	(177.7, 233.7)	(62.9, 73.4)	(24.5, 36.7)	(26.0,6, 36.6)	(68.5, 76.0)

S-Table 3: Model comparison of negative regression analysis

Variable	IRR (95% CI) p-value			
	Model 2	p-value	Model 3	p-value
**Age group, years**				
<3	0.96 (0.79, 1.16)	0.564	0.96 (0.79, 1.16)	0.556
4-6	1.39 (1.22, 1.59)	**<0.001***	1.39 (1.22, 1.59)	**<0.001***
7-12	1.47 (1.34, 1.61)	**<0.001***	1.47 (1.34, 1.61)	**<0.001***
13-18	1.50 (1.36, 1.65)	**<0.001***	1.50 (1.36, 1.65)	**<0.001***
19-64	1 (Reference)		1 (Reference)	
65-74	0.72 (0.59- 0.89)	**<0.001***	0.73 (0.59- 0.89)	**<0.001***
>=75	0.77 (0.64, 0.92)	**<0.001***	0.77 (0.64, 0.92)	**<0.001***
**RDT results**				
Flu A	1 (Reference)		1 (Reference)	
Flu B	0.84 (0.77, 0.90)	**<0.001***	0.84 (0.78, 0.91)	**<0.001***
Not attributed	0.70 (0.48, 0.98)	**0.014**	0.70 (0.49, 1.00)	**0.014**
**Vaccination history**				
No	1 (Reference)		1 (Reference)	
Yes	1.08 (0.97, 1.19)	0.088	1.08 (0.97, 1.19)	0.087
Unknown	1.13 (0.98, 1.31)	**0.042**	1.13 (0.98, 1.31)	0.042
Mean household size	1.18 (0.71, 1.86)	0.411	1.20 (0.74, 1.94)	0.376
**Population per district**				
<500	1 (Reference)		1 (Reference)	
500-2000	2.31 (2.04, 2.63)	**<0.001***	2.31 (2.04, 2.63)	**<0.001***
>2000	4.41 (3.81, 5.14)	**<0.001***	4.42 (3.81, 5.14)	**<0.001***
**Vaccination coverage**				
<50	1.10 (0.85, 1.43)	0.363	1.10 (0.84, 1.42)	0.375
50-60	1 (Reference)		1 (Reference)	
60-65	0.97 (0.89, 1.06)	0.423	0.97 (0.89, 1.06)	0.416
>65	0.76 (0.66, 0.88)	**<0.001***	0.76 (0.66, 0.88)	**<0.001***
**Proportion unvaccinated in neighborhood district**	1.54 (0.74, 3.20)	0.174	1.54 (0.74, 3.20)	0.173
**Influenza season**				
2010/11	1 (Reference)		1 (Reference)	
2011/12	0.83 (0.73, 0.96)	**0.007**	0.83 (0.72, 0.95)	**0.006**
2012/13	0.93 (0.81, 1.07)	0.296	0.93 (0.81, 1.06)	0.263
2013/14	0.87 (0.77, 0.99)	**0.039**	0.88 (0.72, 1.00)	**0.042**
2014/15	0.82 (0.72, 0.94)	**0.003**	0.83 (0.72, 0.95)	**0.005**
2015/16	1.13 (0.94, 1.35)	0.159	1.16 (0.96, 1.40)	0.104
2016/17	0.81 (0.70, 0.94)	**0.004**	0.82 (0.71, 0.95)	**0.006**
2017/18	1.09 (0.92, 1.29)	0.273	1.11 (0.94, 1.33)	0.184
**Seasonality**				
Sin	1.00 (0.95, 1.05)	0.600	NA	NA
Cosin	1.01 (0.96, 1.06)	0.619	NA	NA

Results of regression analysis after we changed the population per district and vaccination coverage to categorical variables (Model 2 and Model 3), after removal of control for seasonality (Model 3)

S-Table 4: Seasonal influenza vaccine coverage by age group (year) for influenza vaccine in the period 2010/11- 2017/18, Kamigoto island, Japan

(A)

Season	<5	5-14	15-64	>65	Total
**2010/11**	79.4	77.1	41.1	72.9	56.0
**2011/12**	83.7	88.2	43.1	72.9	58.0
**2012/13**	81.6	86.7	41.7	70.4	56.3
**2013/14**	84.1	88.2	42.2	68.9	56.4

(B)

SEASON	<3	4-6	7-12	13-18	19-64	65-74	>=75	TOTAL
**2014/15**	77.6	87.7	83.3	74.1	44.7	58.9	78.1	58.6
**2015/16**	86.6	90.1	92.6	84.2	47.5	62.4	78.9	62.1
**2016/17**	69.9	84.5	81.3	75.8	46.7	63.3	78.0	60.0
**2017/18**	78.7	91.1	85.7	83.2	49.8	62.4	77.6	61.1

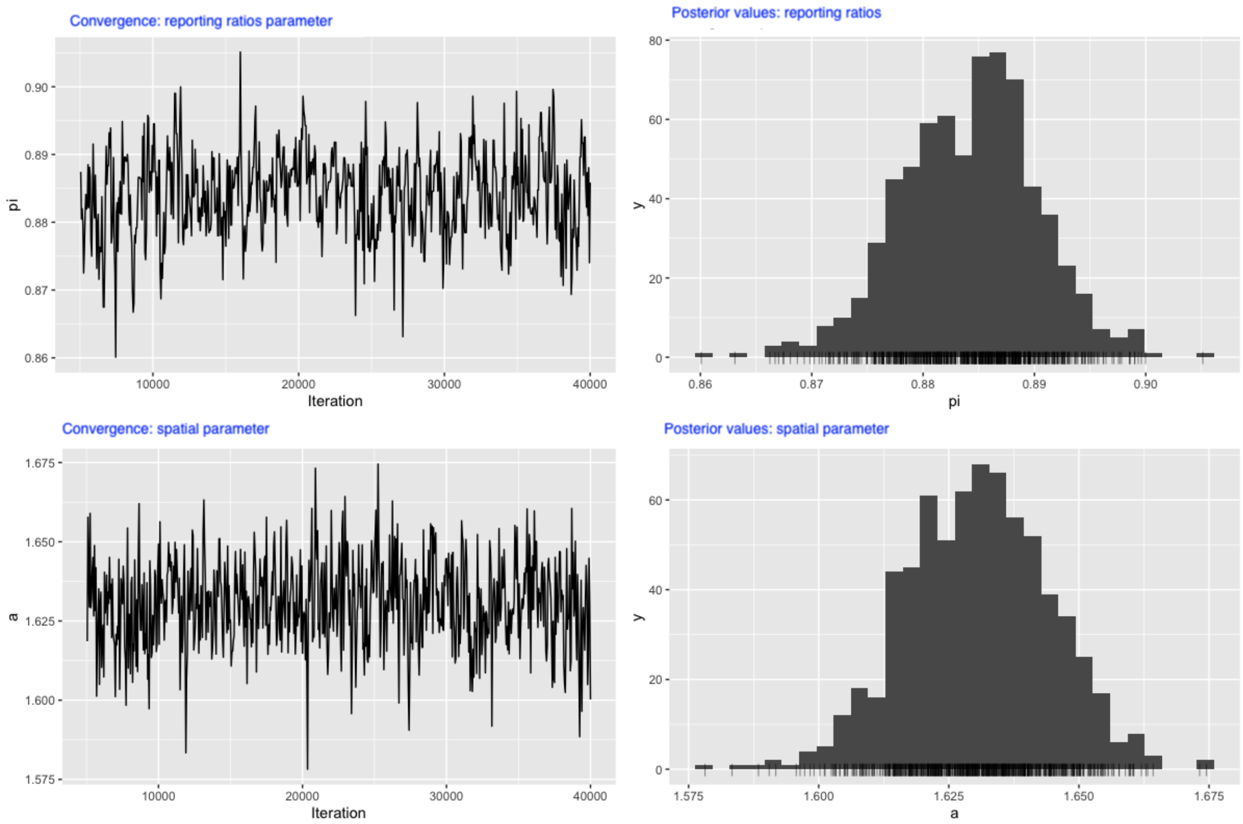

S-Figure 1: Convergence of posteriors and results of posterior estimates

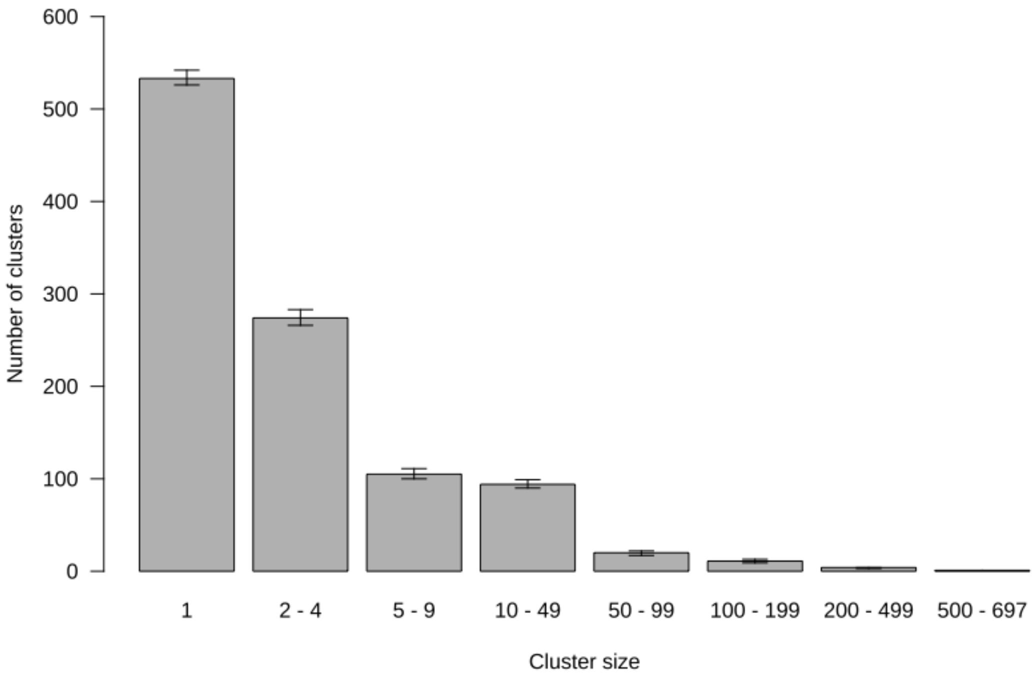

S-Figure 2: Cluster size distribution generated by o2geosocial using the Stouffer rank method

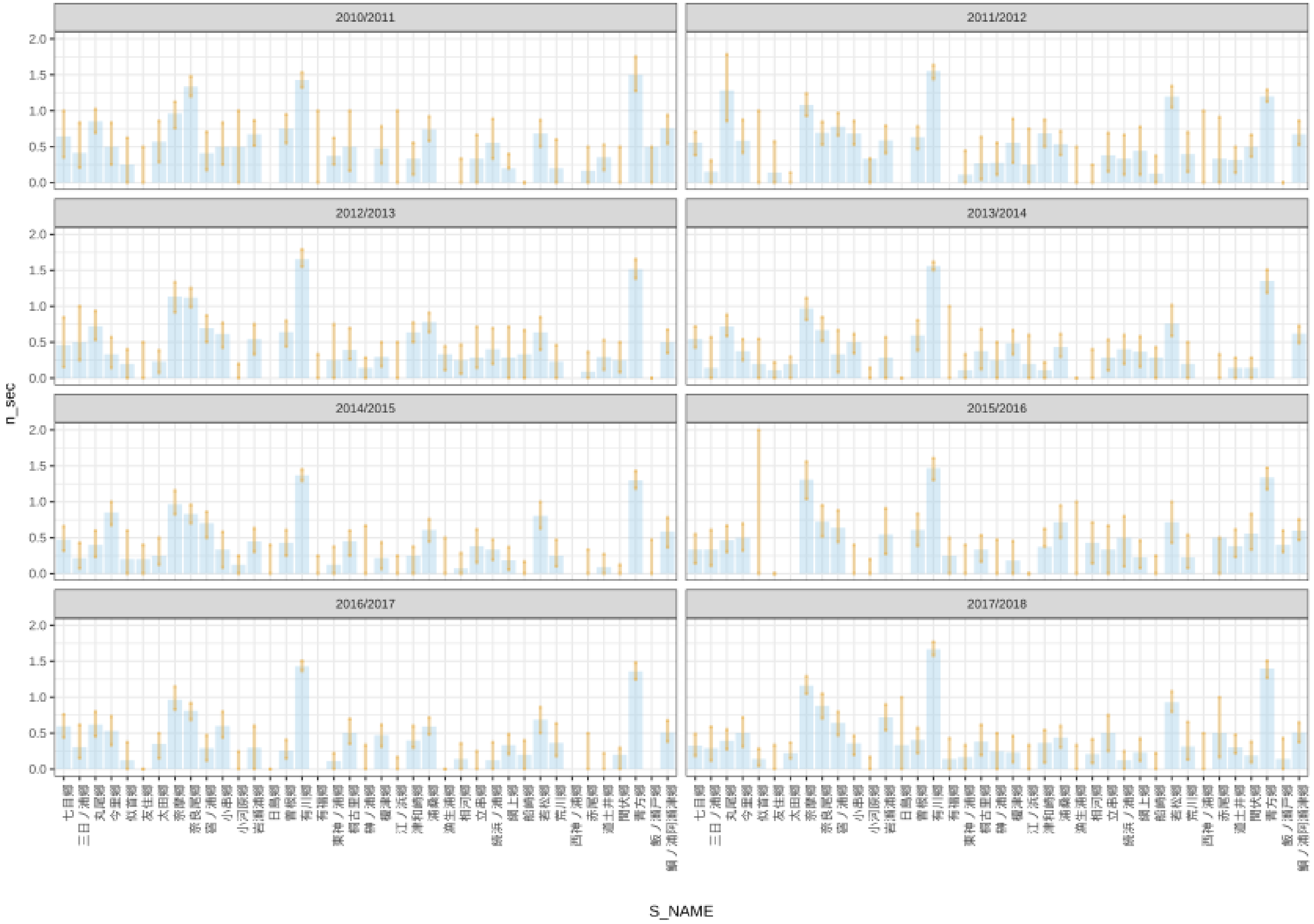

S-Figure 3: Median number of secondary cases per case by district and by season (2010/11- 2017/18)

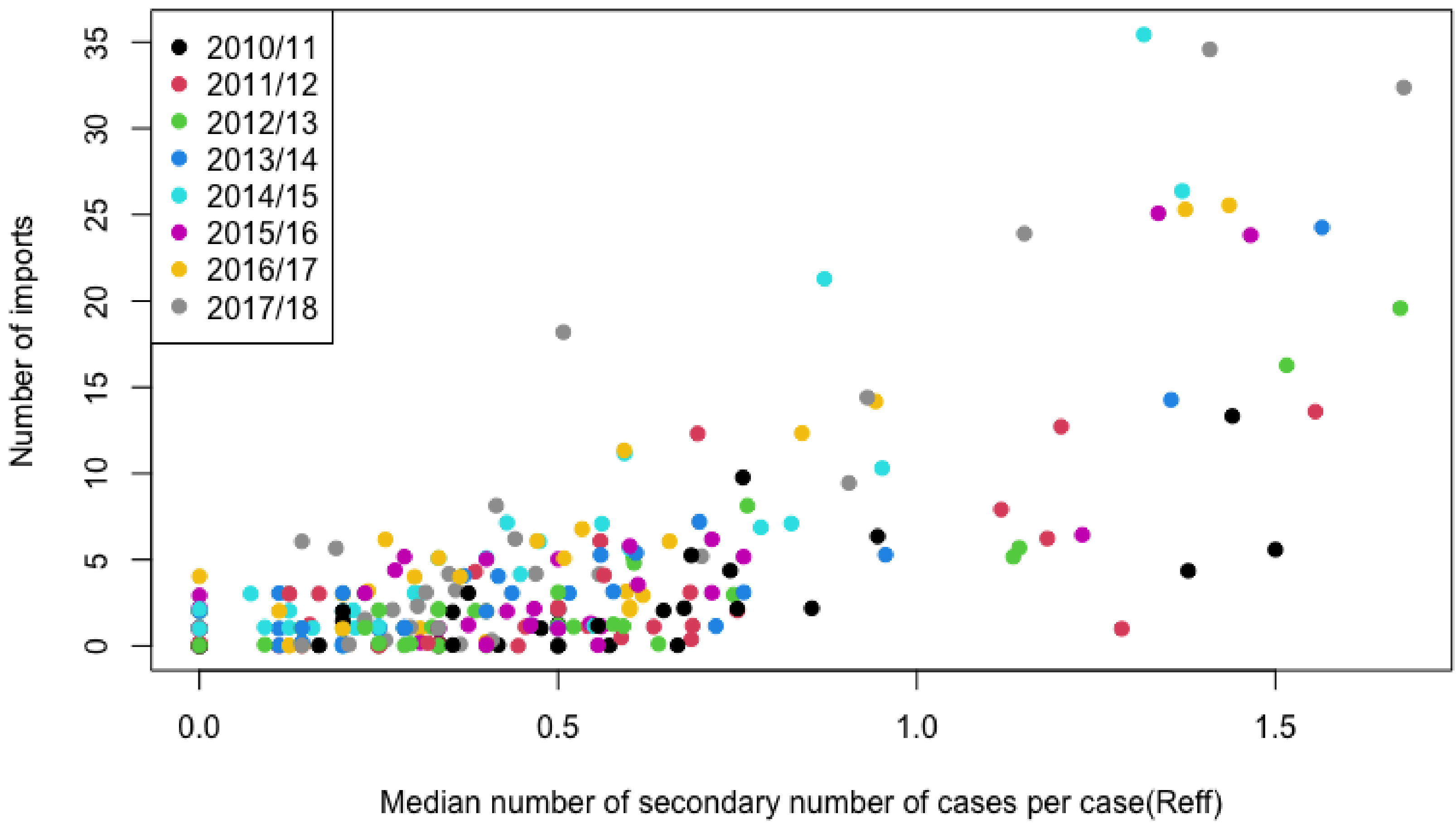

S-Figure 4: The relationship between the median number of imported cases and the median number of secondary cases generated by case in each region, colored by seasons

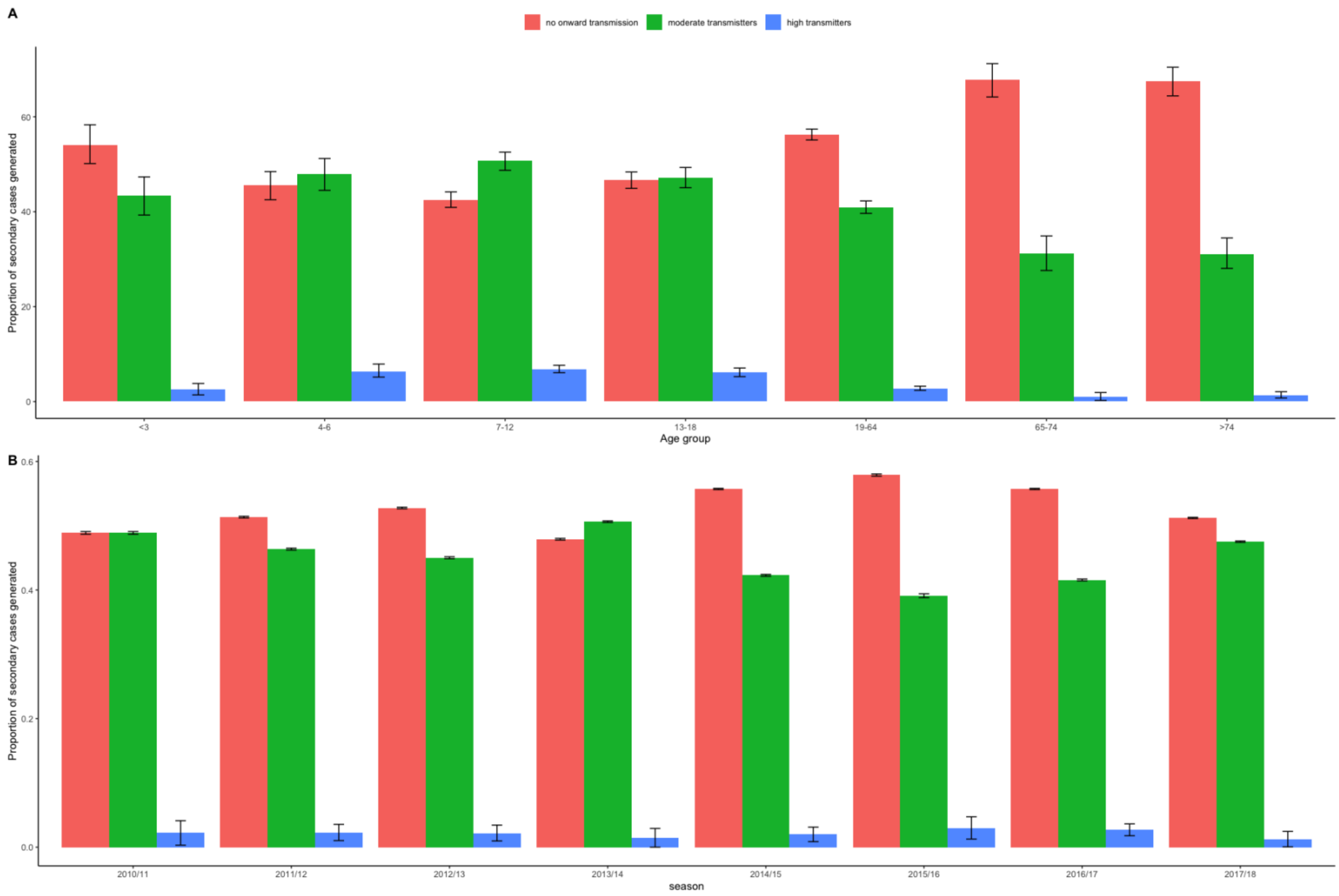

S-Figure 5: Distribution of Reff by age-group and by season

Note: No onward transmission = zero transmission, moderate transmitter = 1-3 secondary cases, High transmitter= more than three secondary cases)

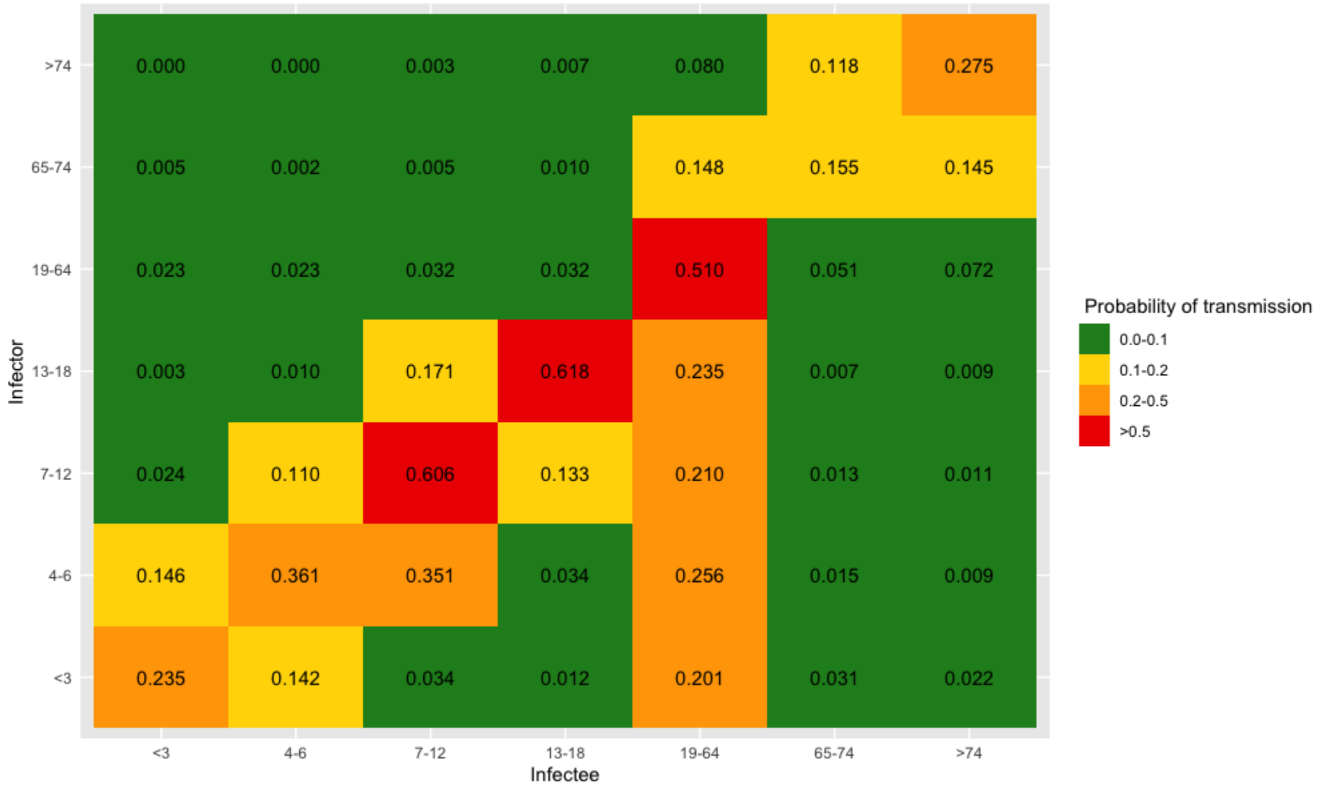

S-Figure 6: Heatmap of the probability of transmission between and within different age groups.

The color of each tile in the heatmap corresponds to proportion of an individual from the age group in each row infecting to another individual from the group in column. The values are extracted from the transmission tress (700 MCMC trees) constructed using o2geosocial package.

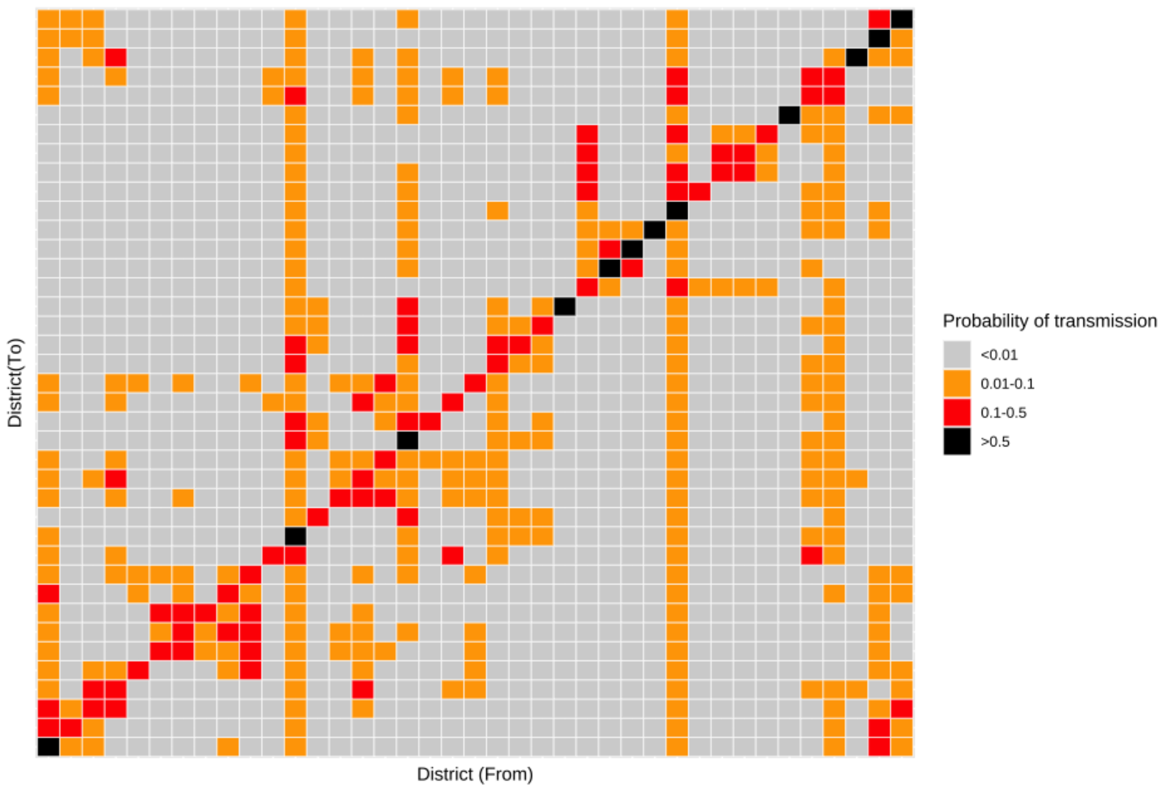

S-Figure 7: Heatmap of the probability of transmission between and within districts
